# Dye-free visualisation of intestinal perfusion using laser speckle contrast imaging in laparoscopic surgery: a prospective, observational multi-centre study

**DOI:** 10.1007/s00464-023-10493-0

**Published:** 2023-10-09

**Authors:** Wido Heeman, Joost Calon, Arne van der Bilt, Jean-Pierre E. N. Pierie, Ilona Pereboom, Gooitzen M. van Dam, E. Christiaan Boerma

**Affiliations:** 1 Faculty Campus Fryslân, University of Groningen, Wirdumerdijk 34, 8911 CE Leeuwarden, The Netherlands; 2 Department of Surgery, University Medical Centre Groningen, 9713 GZ Groningen, The Netherlands; 3 LIMIS Development BV, 8934 AD Leeuwarden, The Netherlands; 4 ZiuZ Visual Intelligence BV, 8401 DK Gorredijk, The Netherlands; 5 Medical Center Leeuwarden, Department of Surgery, 8934 AD Leeuwarden, The Netherlands; 6 Post Graduate School of Medicine, University Medical Center Groningen, 9713 GZ Groningen, The Netherlands; 7 Department of Surgery, Nij Smellinghe Hospital, 9202 NN Drachten, The Netherlands; 8 Department of Intensive Care, Medical Center Leeuwarden, 8934 AD Leeuwarden, The Netherlands

**Keywords:** Laser speckle contrast imaging, Perfusion, Laparoscopic, Intestinal perfusion

## Abstract

**Introduction:**

Intraoperative perfusion imaging may help the surgeon in creating the intestinal anastomoses in optimally perfused tissue. Laser speckle contrast imaging (LSCI) is such a perfusion visualisation technique that is characterized by dye-free, real-time and continuous imaging. Our aim is to validate the use of a novel, dye-free visualization tool to detect perfusion deficits using laparoscopic LSCI.

**Methods:**

In this multi-centre study, a total of 64 patients were imaged using the laparoscopic laser speckle contrast imager. Post-operatively, surgeons were questioned if the additional visual feedback would have led to a change in clinical decision-making.

**Results:**

This study suggests that the laparoscopic laser speckle contrast imager PerfusiX-Imaging is able to image colonic perfusion. All images were clear and easy to interpret for the surgeon. The device is non-disruptive of the surgical procedure with an average added surgical time of 2.5 min and no change in surgical equipment. The potential added clinical value is accentuated by the 17% of operating surgeons indicating a change in anastomosis location. Further assessment and analysis of both white light and PerfusiX perfusion images by non-involved, non-operating surgeons showed an overall agreement of 80%.

**Conclusion:**

PerfusiX-Imaging is a suitable laparoscopic perfusion imaging system for colon surgery that can visualize perfusion in real-time with no change in surgical equipment. The additional visual feedback could help guide the surgeons in placing the anastomosis at the most optimal site.

For colon cancer, the third most prevalent malignancy worldwide, radical surgical resection remains the cornerstone of treatment. Although various improvements in surgical (minimally invasive) techniques, perioperative care and patient optimisation strategies have led to a reduced risk of surgical complications, anastomotic leakage still remains a feared and potential lethal complication [1].

Most risk factors, such as smoking, body mass index, sex and premorbid conditions, cannot be controlled by the surgical team [2–4]. However, (in)adequate anastomotic perfusion is a potentially modifiable and preventable risk factor, and has been identified as a prerequisite for uncompromised anastomotic healing [5–7]. Insight in anastomotic perfusion has gained major interest in recent years. Enabling the surgeon to assess and—if necessary and possible—relocate the anastomosis to ensure adequate perfusion, which potentially may result in a decrease in anastomotic leakage [8–12].

The current gold standard for perfusion assessment, a mere visual inspection, is limited to subjective indicators of intestinal viability such as tissue discoloration, pulsatile motion and bleeding of the resected edges [13]. This emphasizes the need for a more objective and ideally quantitative measure of intestinal perfusion, enabling surgeons to determine the optimal site for the anastomosis. The method should preferably be suitable for laparoscopic use, robust and non-invasive [14]. Laser speckle contrast imaging (LSCI) is a novel perfusion imaging method that has recently been demonstrated in the setting of open- [15–18] and laparoscopic-colon surgery [19–22]. It was able to instantaneously distinguish between poor- and well-perfused colon tissue without the need of a contrast agent [23]. In this prospective observational clinical feasibility study, LSCI with PerfusiX-Imaging was used to image intestinal perfusion. The aim of the current trial is to study to what extent real-time intraoperative clinical decision making is influenced by the addition of PerfusiX-Imaging perfusion maps compared to the standard of practice in colorectal surgery.

## Methods

### Patients

This prospective, observational, multi-centre study was performed in two regional hospitals in the Netherlands (i.e., Medical Centre Leeuwarden and Nij Smellinghe Hospital Drachten). For this study, a total of 67 patients that underwent an elective right-sided hemicolectomy, left-sided hemicolectomy or sigmoid resection between February 2021 and April 2022 were included. In total, 64 imaging procedures were performed. This study was conducted according to the ethical principles stated in the Declaration of Helsinki. Written informed consent was obtained from every patient prior to inclusion. The study was approved by the local ethics committee (NL74681.099.20) and registered in the World Health Organization public trial registry (trialsearch.who.int, NL9215).

### Laparoscopic LSCI system PerfusiX-Imaging

The laparoscopic LSCI device used in this study is PerfusiX®-Imaging system (LIMIS Development BV, Leeuwarden, The Netherlands), specifically designed to allow for connectivity with a range of currently existing available laparoscopic video systems. In this study, the investigational medical device was used in conjunction with a standard Olympus laparoscopic video system and a chip-on-the-tip laparoscope (EndoEye®, Olympus, Hamburg, Germany). PerfusiX-Imaging provides instantaneous, continuous and repetitive real-time perfusion images of the intestine without the use of a contrast agent. Perfusion maps were displayed on a separate display, blinded to the surgeon during the surgical procedure. The device uses a red laser light source to homogenously illuminate the tissue of interest. Due to random interference, a so-called speckle pattern is formed. The laser speckle technology is based on the principle that the speckle pattern changes when the tissue contains moving particles (i.e., red blood cells) [23]. The rate of change corresponds to the perfusion.

### Surgery and imaging procedure

Surgical procedures were executed according to the standard-of-care without any interference. The surgeons did not have access to the acquired images during the procedure. Image acquisition was performed before intestinal resection at the proximal and distal intestine transection before intestinal transection, which make up the afferent and efferent parts of the subsequent intestinal anastomosis, as indicated by the operating surgeon. Subsequently, the surgeon(s) were asked to indicate the exact location and orientation of the anastomosis. The perfusion images were displayed to the researcher in real time, but blinded to the surgeon(s) during the procedure. The total added operating time for the recording of four imaging sets was recorded in minutes. One imaging set consisted of an LSCI and white light image of the proximal and distal transection locations.

### Evaluation of laser speckle perfusion images

The surgeons were shown one training image to help understand the perfusion images. The images are displayed in the scientifically substantiated colourmap Viridis to minimize observer bias as a result of colour perception [24]. Perfusion images were shown to the operating surgeon in the immediate postoperative phase. Later on, images were also assessed by non-involved, non-operating surgeons to study inter-observer variability. Additionally, to assess the intra-observer variability, the operating surgeons were confronted with the same perfusion images for a second time in a blinded, randomized order to re-assess the initial decision. The operating and non-operating surgeons were asked if the images were clear and whether they would have changed the location of the anastomosis based on the PerfusiX-Imaging derived perfusion images (yes/no). The indicated change in location was noted (in centimetres). Lastly, the homogeneity of the decision to change the location between non-operating surgeons was examined.

### Statistical analysis

All data were analysed using SPSS (SPSS Inc., Version 28.0.1, Chicago, United states). Data are represented as mean ± standard deviation, unless stated otherwise. Descriptive statistics included measurements of distribution such as (geometric) means with standard deviation, medians with range and frequencies with 95% confidence interval. Applicable parametrical tests were used and a two-sided *p*-value of *p* < 0.05 was considered statistically significant.

## Results

### Patient and procedural characteristics

**Fig. 1:**
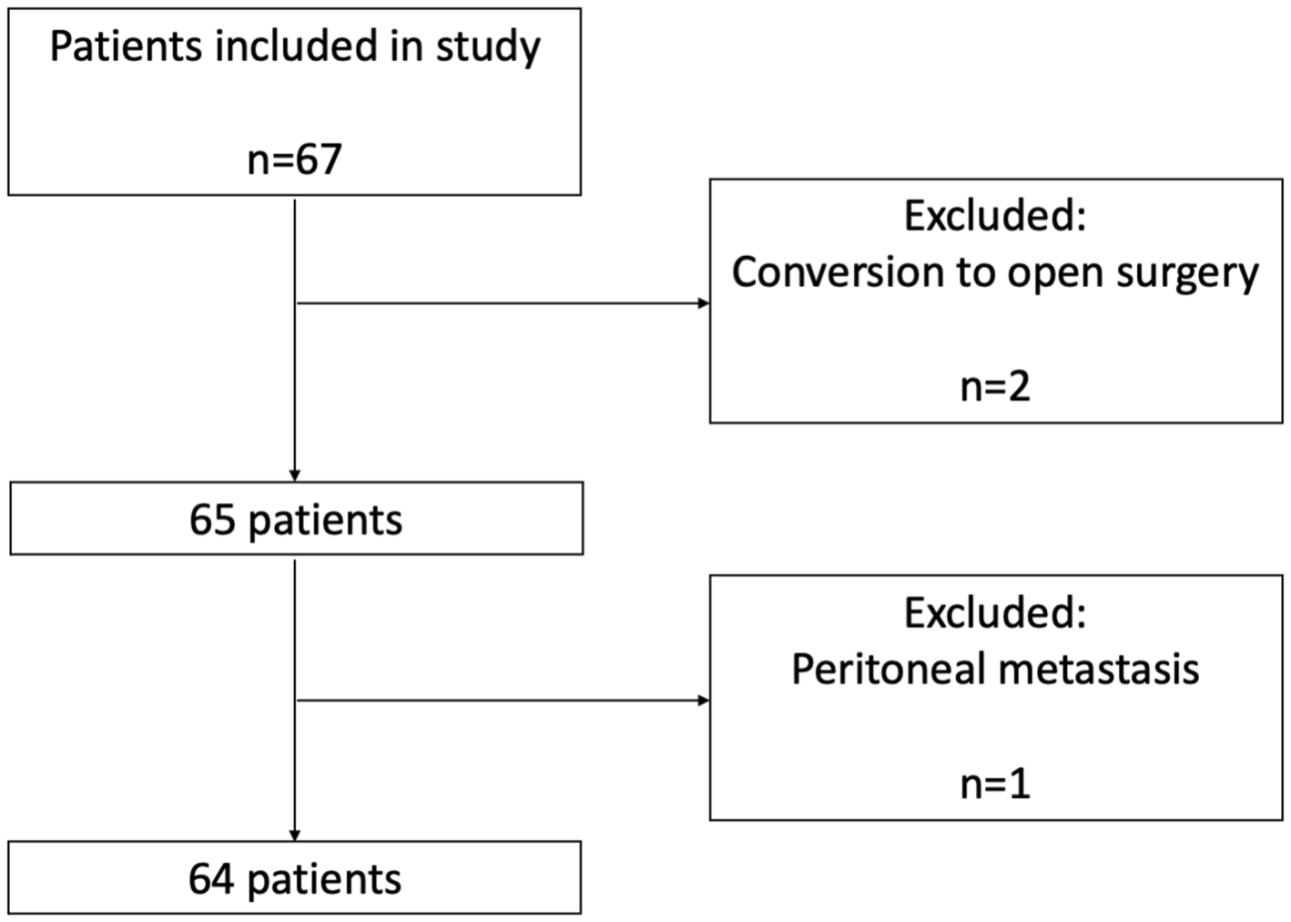
Flow diagram of patient inclusion

Patient characteristics are presented in Table 1. Sixty-seven patients were enrolled in the study and 64 imaging procedures were performed. Two patients were excluded afterwards due to conversion to open surgery and one patient due to the presence of peritoneal metastasis and subsequent termination of the procedure (Fig. 1). In total 42 (66%) patients underwent a right-sided hemicolectomy, 4 (6%) patients a left-sided hemicolectomy and 18 (28%) patients underwent a sigmoid resection. Two patients (3.1%) developed anastomotic leakage. No adverse events were associated with the imaging device. The average extra surgery time due to the use of the PerfusiX-Imaging system was 2.5 min (range 2–5).

**Table 1:** Demographic and clinical characteristics of patients

Characteristics	*n* (%)
Total patients	64
Males	34 (53%)
Females	30 (47%)
Age (years)	
Mean	67
Median	68
Range	32–90
BMI	
Mean	26
Median	26
Range	19–36
Type of procedure	
Right-sided hemicolectomy	42 (66%)
Left-sided hemicolectomy	4 (6%)
Sigmoid resection	18 (28%)
Anastomotic leakage	2 (3.1%)

*BMI* body mass index

### Laparoscopic LSCI perfusion assessment

**Fig. 2:**
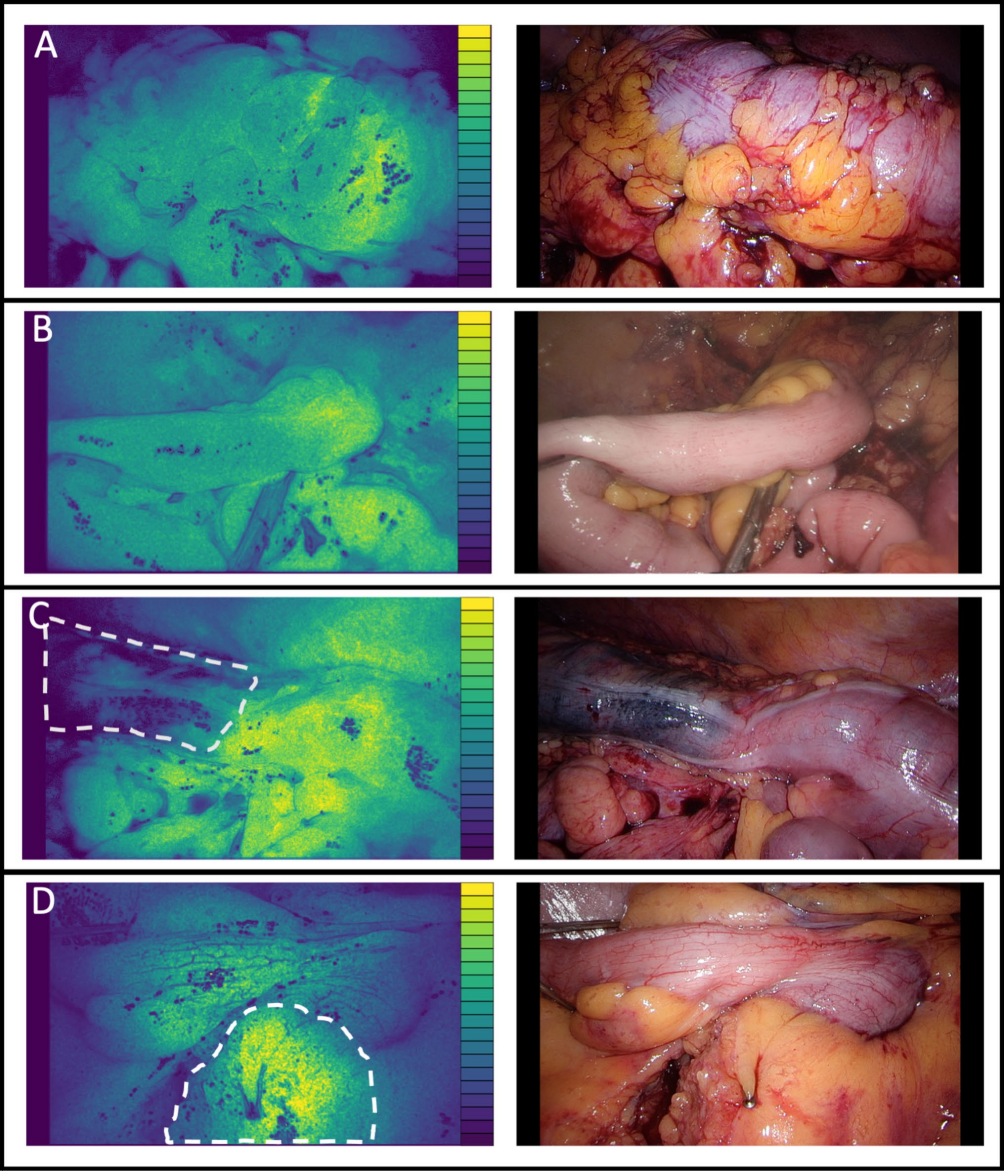
**A** Perfusion map of the large intestine indicating an ischemic area on the proximal side and white light image of the large intestine with slight tissue discoloration on the proximal side. The scale bar indicates high flow (yellow) and low flow (dark blue). **B** Perfusion map of the small intestine indicating an ischemic area on the distal side and white light image of the small intestine with no clear indications of ischemic tissue. **C** Perfusion map of colon tissue with an imaging artefact caused by tumour marker ink and white light image of the colon with tumour marker ink on the proximal side. The tumour marker ink is highlighted. The difference in optical properties caused by the ink falsely gives the impression of ischemic tissue. **D** Perfusion map of the small intestine with an imaging artefact caused by fatty tissue. The fatty tissue is highlighted. The difference in optical properties between the small intestine and fat gives the false impression that the fatty tissue has high blood flow and white light image of small intestine indicating with fatty tissue (Color figure online)

Real-time visualization of intestinal perfusion was successfully achieved in all imaging procedures resulting in interpretable perfusion images. In 39% of the cases relative ischemia was inspected by the naked eye of the surgeon and confirmed by the perfusion images. In these cases, ischemic tissue was visible in white light images (Fig. 2a) that was confirmed by the speckle images. In other cases, there was no indication of ischemic tissue in the white light images (Fig. 2b) whereas this was the case in the speckle images. The colonic perfusion could generally be split up in three sections; well perfused, disturbed perfusion and poor perfusion (Fig. 2a and b). The disturbed area was small in the watershed area between well- and poor perfused tissue. Perfusion measurements were affected by the marker ink (Fig. 2c) and fatty tissues (Fig. 2d).

### Operating surgeon

Operating surgeons indicated to change the location of the anastomosis in 17% (*p* < 0.001) of the cases after reassessment of the site of anastomosis based on the additional PerfusiX-Imaging-derived visual feedback. The estimated change in the location towards the proximal or distal side was 1.2 cm on average (range 0.5–2 cm). None of the operating surgeons indicated that the images were unclear or difficult to interpret.

Intra-observer variability by reassessment of the operating surgeon showed that they were in agreement with their own clinical decision making in 81% of the cases. If the operating surgeon first indicated no change in location, in 84% of the cases the blinded operating surgeons also indicated no change in location. If the operating surgeon initially indicated a change in location, in 67% of the cases the blinded operating surgeons also indicated a change in location.

Two patients developed anastomotic leakage. One anastomotic leak was caused by a structural defect for which the patient was re-operated within 24 h. For this patient the operating surgeon postoperatively did not indicate a change in location of the anastomosis based on the additional visual feedback. The second anastomotic leak was detected 4 days postoperatively. In this case, the surgeon indicated postoperatively a change in location of the anastomosis.

### Non-operating surgeons

A total of six non-involved, non-operating surgeons were shown all 64 imaging sets. Prior to the assessment, five surgeons had performed surgery with PerfusiX-Imaging at least three times with an average of fourteen imaging sessions. One surgeon had no hands-on experience with the PerfusiX-Imaging device. The non-operating surgeon would have changed the location of the anastomosis in 22% (*p* < 0.0001) of the cases based on the PerfusiX-Imaging derived visual information. The estimated change in the location towards the proximal or distal side was 1.3 cm on average (range 0.5–2 cm). The surgeons indicated that they did not feel secure to answer the question based solely on the white light and perfusion images in 10% of the cases, of which 43% were sigmoid resections and 57% right-sided hemicolectomy. As for the anastomotic leakage patients, the non-operating surgeons indicated no change in location of the anastomosis caused by the structural defect. On the other hand, similar to the operating surgeon, the non-operating surgeons indicated a change in location of the anastomosis in the other anastomotic leak.

### Comparison of operating and non-operating surgeons

The percentage of anastomoses indicated to be relocated and the change in location in centimetres between operating and non-operating surgeon groups did not differ significantly (17% vs. 22%). Overall, the non-operating surgeons were in agreement with the operating surgeons in 80% of the cases. If the operating surgeon indicated no change in location, in 81% of the cases the non-operating surgeons also indicated no change in location. If the operating surgeon indicated a change in location, in 71% of the cases the non-operating surgeons also indicated a change in location. The percentage of non-operating surgeons that did not feel secure to answer the question solely on perfusion images was significantly higher than the operating surgeons (10% vs. 0%, *p* < 0.05). In 20% of the cases both operating and individual non-operating surgeons were unanimous. In all of these cases, no adjustments were advised to the procedure performed.

## Discussion

We report on the use of intraoperative laparoscopic LSCI perfusion assessment in intestinal surgery. The data of this study indicate that surgeons would relocate the anastomosis based on the additional PerfusiX-Imaging derived visual feedback in a significant number of patients. The consonance between the operating and non-operating surgeons is indicative for the objectivity and clinical value of the technology. Yet, the differences in interpretation between surgeons suggest that PerfusiX-Imaging, in combination with current intraoperative indicators of intestinal viability, could be of added value in clinical decision making, rather than a dichotomous stand alone. The relatively small adjustments in the amount of resected bowel are highly unlikely to affect functional patient outcome, however, they might be required to reduce clinically overt anastomotic leakage.

All imaging procedures were successful and surgeons were able to correctly interpret the images in all cases. The little added operating time is indicative for the ease of integration into the surgical workflow. The presented laser speckle technology is capable of visualizing intestinal perfusion in real time. The images display clear perfusion differences to the surgeon without the need for exogenous contrast. The watershed area, present in several cases, indicates the necessity for precise perfusion monitoring to position the anastomosis and intestinal transection in well-perfused tissue.

Perfusion assessment by means of imaging does not have any therapeutic effect by itself, it is the surgeons’ job to properly perform the imaging, to correctly interpret the images, and to act upon it in a sensible way [25]. Perfusion images from PerfusiX-Imaging were indicated as clear by all operating surgeons. However, the user has to be aware of imaging artefacts when interpreting the perfusion images. For example, the optical effect of large amounts of fat or tumour marker ink. These imaging artefacts can be attributed to the difference in scattering and absorption compared to colonic tissue.

The operating and non-operating surgeons’ behaviour with respect to the indication of change in location of the anastomosis, as well as the total change in location in centimetres, does not differ statistically. However, the non-operating surgeons have a significantly higher percentage of cases that are uncertain solely based on the perfusion imaging. This underlines the use of optical imaging complementary to, and not as a replacement of, the surgeon’s visual perfusion assessment. The addition of perfusion imaging will likely improve the surgeons’ judgement in combination with intraoperative observations and thereby might decrease morbidity and mortality. Even though the data of this study suggest that most relocations are not likely to have prevented anastomotic leakage because of the low overall leakage rate (3%), it seems cogent that selecting the optimal site of an anastomosis, could help prevent anastomotic leakage its sequalae.

The interest in perfusion imaging in colorectal surgery was boosted with the recent influx of FDA approved laparoscopic fluorescence imaging devices. Several clinical trials speculate that anastomotic leakage rates decrease after anastomotic perfusion imaging [8]. However, conclusive results are only achieved in a recent meta-analysis [26] due to the high patient numbers required for adequate statistical power [6, 8, 27]. Although indocyanine green fluorescence imaging and LSCI seem to have a similar clinical applicability, laser speckle has several advantages as described by others [28, 29]. Yet, studies comparing the effect of LSCI and indocyanine green fluorescence imaging on anastomotic leakage is still lacking. LSCI allows the surgeons to perform repetitive and continuous real-time imaging at different time points during surgery without the need for intravenous administration of fluorescent contrast [28, 29]. The imaging timeframe of LSCI is limitless whereas for indocyanine green angiography this is very limited due to clearance of the contrast. LSCI measures the actual movement of red blood cells compared to the concentration of fluorescent dye bound to blood proteins in indocyanine green angiography. This means that LSCI signal represents the flow of red blood cells rather than the concentration of blood proteins. Our laparoscopic LSCI data are comparable to another study where the actual transaction line was compared with the LSCI transection line [15]. Also, others already showed that it is possible to image colonic perfusion with this LSCI in an open setting with similar images [16]. Last, the same group performed a small intervention study with a large reduction anastomotic leakage compared to a historical group [17].

When assessing the perfusion in the distal rectosigmoidal transection site in patients undergoing a sigmoid resection, results were uncertain in a proportion of the cases. This might be related to the limited sub-surface measurement depth of LSCI in combination with the increasing sigmoid mesocolon folding around the sigmoid. This causes less colon tissue to be visible, thereby decreasing the surgeons’ confidence in the perfusion images. Whether this might indicate that LSCI is less suitable for perfusion measurements during lower sigmoid resections and rectal surgery remains to be established.

Due to the prospective and observational nature, these data can only lead to speculative conclusions regarding anastomotic leakage. Stronger evidence based on larger patient numbers is needed to prove the effectiveness of LSCI in the reduction of anastomotic leakage. Preferably, a randomized control trial should be conducted to study the effect on the anastomotic leakage incidence. Based on our experience, the real-time video imaging for an intervention study should be performed when both stumps are created. The additional perfusion feedback then provides the surgeons with valuable information at this stage as the perfusion is not further altered whilst the surgeon can still adjust the site of the anastomosis. Additionally, the anastomosis could be imaged for a complete anastomotic perfusion assessment.

## Conclusion

In this multi-centre observational study, the additive use of PerfusiX-Imaging during colon surgery was associated with a significant percentage of both operating and non-operating surgeons to relocate the anastomosis to a more optimally perfused region. The effect of PerfusiX-Imaging on anastomotic leakage should be further investigated in a large clinical trial. Also, the ability to measure perfusion unintrusive to the surgical procedure potentially has many more clinical applications that should be explored in clinical trials.
